# A novel technique of penetrating keratoplasty to prevent intraocular contents extrusion for infectious keratitis

**DOI:** 10.1186/s12886-023-03025-w

**Published:** 2023-07-17

**Authors:** Yukun Yang, Qian Kang, Hao Lian, Wei Qi, Duanrong Cao, Xiaoming Yao

**Affiliations:** 1 Chengdu Aidi Eye Hospital, 45, Sec.2, West Second Ring Rd, Chengdu, 610072 Sichuan China; 2 Shenzhen Huaxia Eye Hospital, Lianhua Road 2032-1, Shenzhen, China; 3 The People’s Hospital of Baoan District, Shenzhen, 518101 China

**Keywords:** Penetrating keratoplasty, Infectious keratitis, Intraocular contents, Extrusion, Lens-iris diaphragm, Plastic sheet

## Abstract

**Purpose:**

To evaluate the safety and the effectiveness of our novel penetrating keratoplasty for infectious keratitis.

**Methods:**

Retrospective, noncomparative, interventional case series of patients with infectious keratitis who received the novel penetrating keratoplasty technique were analyzed. A prepared plastic sheet was located between the diseased cornea and iris-lens diaphragm. After the diseased lesions were removed, the graft was positioned on the plastic sheet and sutured to the recipient bed. The plastic sheet was pulled out from the anterior chamber before the all interrupted sutures were placed. The intra- and post-operative complications, the outcome of the graft and the number of corneal endothelial cells were analyzed.

**Results:**

A total of 82 eyes of 82 patients was included. The mean follow-up period was 29 ± 16 months (range from 13 to 45 months). No intraocular content extrusion, simultaneous cataract extraction and suprachoroidal hemorrhage occurred. Direct contact between the infectious cornea and the graft was successfully avoided. Greater than expected endothelial cell reduction or complications were not found.

**Conclusions:**

This modified technique effectively prevents the extrusion of intraocular contents while avoiding the direct contact with donor endothelium during the procedure. The occurrence rate of complications such as endothelial cell loss is not higher than the conventional methods.

## Introduction

Suprachoroidal hemorrhage is one of the most devasting intraoperative complications. It occurs more frequently than might be appreciated in corneal transplant, ranging from 0.45 ~ 1.08%.[1, 2] It’s incidence in penetrating keratoplasty is 0.56% with general anesthesia and 4.3% with local anesthesia [1, 2].

Other contributing factors include previous surgery, diabetes, hypertension, older age, anticoagulant therapy and vitreous loss. The rupture of long or short posterior ciliary artery is liable to occur while the intraocular pressure drops rapidly in a short time. The best preventive measures are the control of blood pressure and tachycardia, and especially to avoid the sudden release of aqueous humor during the surgery.

Various modifications have been reported to prevent the forward movement effectively by supplying counterbalance against the extrusion. The most common precautionary measures are to place the graft on the diseased cornea and suture the donor to the recipient bed, then followed by removing the diseased cornea before the rest interrupted sutures was placed [3–5]. It is inevitable that there will be contact between the diseased cornea and the graft. For infectious keratitis, the undesired contact can increase the recurrence of infection [6]. Here, we described a novel technique to avoid rapid forward movement of the iris-lens diaphragm and keep the eye ball stable during removal of diseased corneas, as well as to prevent the direct contact between the infectious cornea and the graft.

## Methods

This retrospective, noncomparative, interventional case series was performed between March, 2017 to March, 2020. It was approved by the Institutional Ethics Committee of Chengdu Aidi Eye Hospital, and performed in compliance with the Declaration of Helsinki. Informed written consent was obtained from all patients. For fungal keratitis, 1% voriconazole and 5% natamycin were used topically, and oral itraconazole (200 mg, once per day) was given if required. For bacterial keratitis, 0.5% Levofloxacin and 0.3% tobramycin were used. Patients who were suitable for deep anterior lamellar keratoplasty or had a lesion extended to the corneoscleral limbus were excluded. Patients with follow-up less than six months were also excluded in the analysis.

### Surgical procedure

**Fig. 1:**
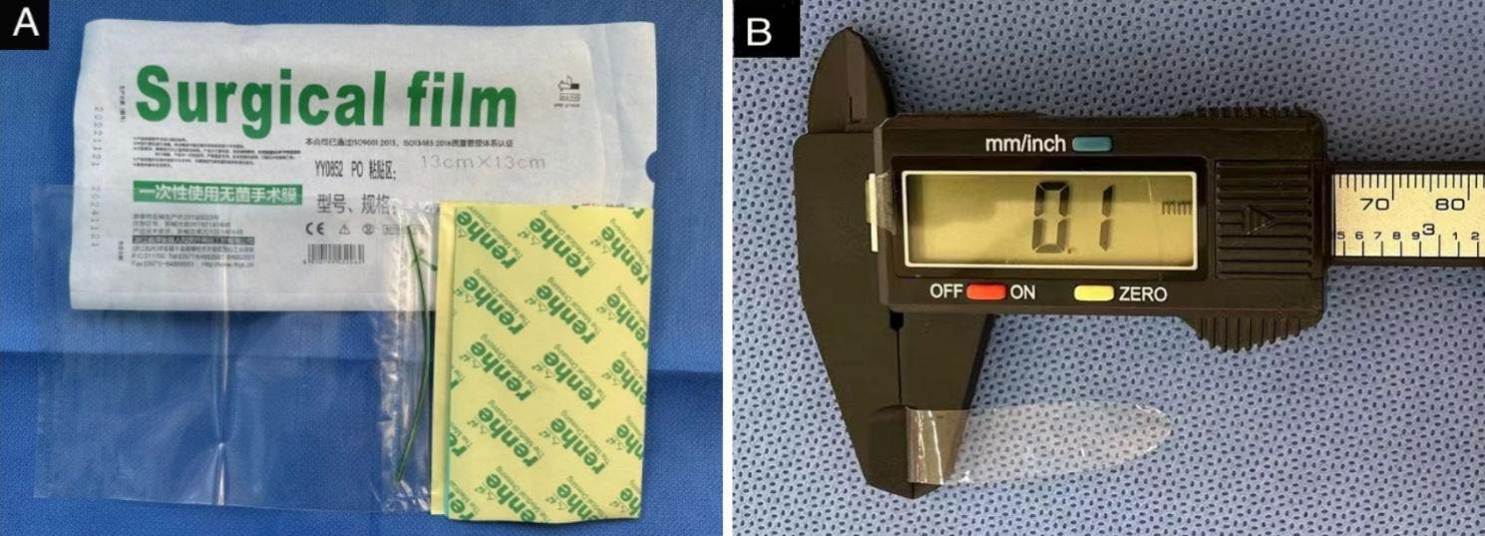
Illustration of the plastic sheet. **A**. Surgical film with drainage bag is used as a protective sheet for the extrusion of intraocular contents. **B**. The sheet is single-layered and the thickness is 0.1 mm

**Fig. 2:**
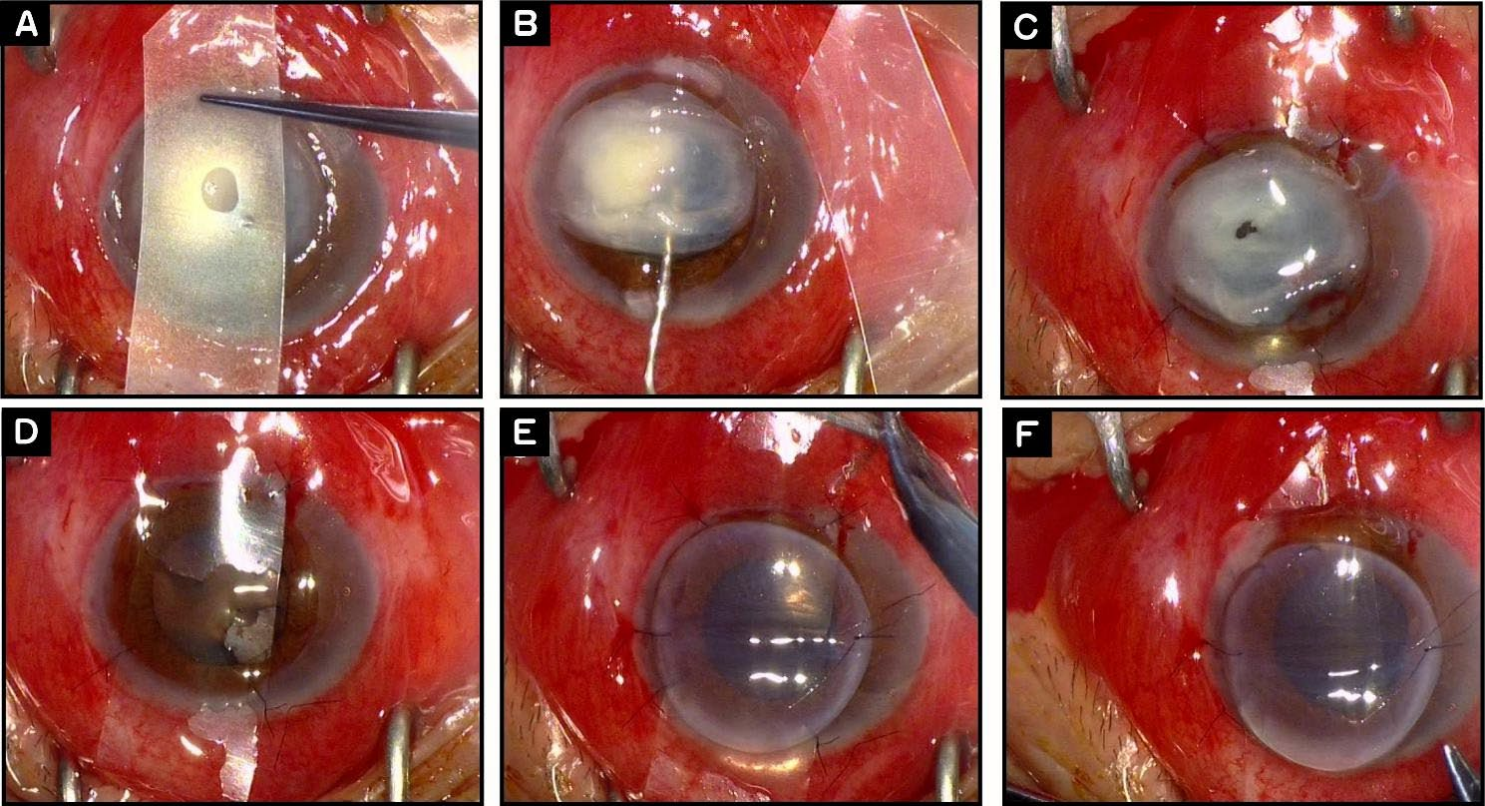
Illustration of the PK technique. **A**. Preparation of the plastic sheet. **B**. Separation of the adhesion between ulcerated cornea and iris-lens with viscoelastic agent. **C**. Insertion of the sheet beneath the ulcerated cornea, fixation of the sheet with four sutures to stabilize the iris–lens diaphragm. **D**. Resection of the ulcerated cornea. **E**. Fixation of the graft with 4 sutures. **F**. Removal of the sutures of the sheet and pulling it out through the superior arc incision secondary by sutures of cornea button

All surgeries were conducted with the modification by four skilled surgeons (X.Y., Y.Y., Q.K., D.C.) under retrobulbar block or general anesthesia. A plastic sheet, approximately 5.0 × 9.0 mm in size (based on the diameter of the trephination) (Fig. 1, A), was cut from the surgical film with drainage bag (Figs. 2 and 100 μm in thickness, Renhe Medical Consumables Inc., Chun’an, Zhejiang, China).

**Fig. 3:**
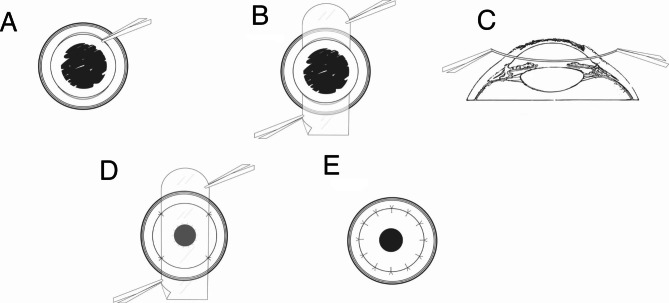
Description of the technique for protecting endothelium and preventing extrusion of intraocular contents with plastic sheet. **(A)** Trephination with suctionless trephine. An adequate size of hand-held trephine is used to make a circular and deep anterior incision to about 2/3 depth of the cornea. Curved corneal scissors used to make two full thickness arc incision from 10 to 2 o’clock and 4 to 8 o’clock in recipient bed. **(B)** Insertion of plastic sheet into anterior chamber. A sheet is cut from drainage bag with 7-7.5 mm wide and 20 mm long and is inserted into anterior chamber, just beneath the ulcerated cornea. **(C)** Sagittal view of plastic sheet inserted beneath the ulcerated cornea. **(D)** The ulcerated cornea is excised, and plastic sheet sutured to the edge of recipient bed could protect the lens-iris diaphragm from protrusion and facilitate the suturing of corneal graft. Note the iris and pupil are visible through the plastic sheet. **(E)** Plastic sheet is removed, and the suture of corneal button is complete

After the partial thickness trephination, two paracentesis were created at the 6- and 12-o’ clock positions, and the full-thickness arc wounds from 4 to 8- and 10-2-o’ clock positions were completed symmetrically. The width of arc wound was based on the size of the plastic sheet. After completing the lamellar demarcation of the recipient bed with trephination, the full-thickness incision of the vertical or horizontal diameter of the recipient bed with its arc length of about 6.75 mm was made, the anterior chamber was injected with antibiotic solution to wash the inflammatory exudates (the type of antibiotic depends on the preoperative etiological diagnosis). Then the sufficient viscoelastic agent was injected into the anterior chamber (AC) and the adhesion between the cornea and the lens–iris diaphragm was separated. One end of the plastic sheet was inserted into the anterior chamber via the superior incision, and the other end was pulled out from the inferior incision with curved toothed forceps, just between the infectious cornea and the iris–lens diaphragm. The sheet was then sutured symmetrically to the recipient bed and fixed with four 10 − 0 nylon sutures. Both ends of the plastic sheet were sutured radially in the stromal layer of the recipient margin, and then the donor cornea was sutured in alignment with the recipient bed with 2–4 stitches. Care must be taken not to tying the first two sutures too tightly to avoid pushing the sheet to the donor endothelium and the increased abrasion. The plastic sheet that was placed between the diseased cornea and the lens-iris diaphragm, like a corneal transplant, was sutured in the anterior stromal layer of the recipient bed in radial pattern. Detailed steps of the technique were shown in Fig. 3.

The ulcerated cornea was excised completely. The exudate membranes, as well as the hypopyon over the iris and pupil, were mechanically removed by the “indirect” force of the viscoelastic agent and forceps. After wash with antibiotics in AC, the viscoelastic-coated donor button with its endothelium down was overlaid on the plastic sheet and fixed at the 3 and 9 o’clock positions with four sutures. All 4 tying knots on the sheet were removed, and the sheet was carefully pulled out from AC (Fig. 1). The other steps of penetrating keratoplasty were as usual. The lens and the endothelium of the graft kept untouched during the procedure. Viscoelastic agent was applied to maintain the space between the sheet and the graft as needed.

### Postoperative treatments

Topical or/and systemic treatment are used to decrease the risk of recurrence of initial microbial infections, retro-donor membrane formation, glaucoma and immunological graft rejection postoperatively. The drug delivery method depended on the condition of the operated eye based on previous literatures or expert consensus [7–12]. The others involve antibiotics eye drops and bandage contact lens, etc. [13] For example, in fungal cases, topical 1% voriconazole and 0.5% levofloxacin were applied, and topical 1% cyclosporine A and bandage contact lens should be immediately started once the donor re-epithelization was achieved. Prednisolone 1% may be used after therapeutic keratoplasty only after 2 weeks if there is no evidence of recurrence of infection on slit lamp or confocal examination. For bacterial cases, topical levofloxacin, 1% cyclosporine A and prednisolone acetate were used for at least 3 months. Prompt symptomatic treatment including immunosuppressants and anti-glaucoma drugs was necessary when elevated intraocular pressure or early rejection occurred. All patients were re-checked at postoperative 1 week, 1 month, 3 month and 6 months, respectively. All the intra- and post-operative complications were documented if any.

## Results

82 eyes from 82 patients (53 male and 29 female) with refractory infectious keratitis underwent penetrating keratoplasty using our method. The mean follow-up period was 29 ± 16 months (range, 13 to 45 months). The mean age was 51 ± 12 years (range, 39 to 63 years). 61 cases were infected with fungi and 21 cases were infected with bacteria. 19 cases had corneal perforation and adherent leukoma to iris. Hypopyon was found in 24 cases. Anterior synechia or adherent leukoma was found in 15 cases, and pupil exudative membrane was found in 14 cases. Cataract was found in seven cases. The interval time between PK (penetrating keratoplasty) and infection was 2–7 weeks.

The mean diameter of the graft was 6.75 mm (range, 5.50 to 7.75 mm). Each procedure was completed without severe intraoperative complications, such as suprachoroidal hemorrhage, vitreous prolapse, lens extrusion, suture placement challenges, and iris–lens damage.

There was no recurrence of infectious keratitis. The anti-fungal and anti-bacterial drugs were used for 6 weeks and 3 weeks, respectively. Anatomical integrity was achieved in all eyes. No cases had choroidal and retinal detachment. Shallow anterior chamber was observed in 6 eyes (7.6%). Among these, there were 3 cases with posterior synechia, and another 3 cases with cataract development. Secondary glaucoma was confirmed in 14 eyes (17%), which was mostly caused by inflammation, but cured by topical anti-glaucoma medications.

Four eyes (6%) developed postoperative cataracts with variable degrees of severity. Graft rejection occurred in 13 eyes (17%) and the neovascularization was found in 6 eyes, but the rest grafts were still clear. The mean postoperative endothelial cell densities decreased from 2439 ± 138 cells/mm^2^ to 1978 ± 225 cells/mm^2^ at 1 month (loss of 19%) and 1829 ± 144 cells/mm^2^ at 3 months (loss of 25%).

## Discussion

During PK procedure, the most devasting complication is expulsive suprachoroidal hemorrhage. If it occurs during the open-sky step of the surgery, it typically leads to the expulsion of intraocular contents. Its occurrence in related to old age, hypertension, high myopia, tachycardia during operation, the presence of anterior chamber IOL, previous hemorrhage in the same eye and Valsalva maneuver, et al. [] Sudden decompression could raise episcleral venous pressure. Long-term hypotony provides more opportunity for scleral collapse and translocation of intraocular structure including iris-lens diaphragm, vitreous or retina.

The conventional steps for reducing these contribution factors include the Flieringa ring, local or general anesthesia and ocular massage combined with intravenous administration of mannitol. Although the different types of anesthesia combined with scleral fixation ring are thought to be safe enough in vast majority of cases, still these can be a potential threat to safety of operation, especially when coughing or bucking related with imperfect general anesthesia or raised orbital pressure to venous outflow associated with retrobulbar anesthesia. The challenge of completely eliminating extrusion of intraocular contents remains unresolved for the lack of counterbalance.

Different modifications have been adopted previously. These improvements had been changed from suturing the graft on the full-thickness diseased cornea to suturing the graft on the anterior lamellar bed [2–5]. The aim of these surgeries is not only to prevent expulsion of intraocular contents, but also to avoid iatrogenic cataract and endothelial cell loss. But for infectious keratitis, this undesired touch can increase the recurrence rate of the infection [16]. Placing the fresh donor directly on the infected cornea may increase the incidence rate of postoperative donor infection although it can also prevent the protrusion of the intraocular contents with open-sky technique to some extent [17].

Our approach worked as an enhancement of the previous techniques. The plastic sheet which acted as a “temporary tamponade”, was fixed with 4 sutures, it can resist the extrusion of intraocular contents by alone or combined with the graft. When hypopyon and inflammatory exudation in anterior chamber are eliminated, the donor then was sutured to the recipient bed. Here, the plastic sheet plays the role of a “transitional lenticule”.

No infection recurred in all cases postoperatively. The incidence of postoperative cataract (6%) and secondary glaucoma (17%) is not higher than that of previous report [18, 19]. The endothelial cells are also well protected (loss at 1 month was 19% in the detected cases and loss at 3 months was 25%), the results are not worse than other related reports [20]. In addition, the plastic sheet is thin and transparent enough so that it benefits intraoperative visualization.

A delicate manoeuvre is required during insertion of plastic sheet beneath the ulcerated cornea especially when the diseased cornea adhered with iris. Rough handling may lead to iridodialysis, anterior lens capsule or Zinn’s ligament injury. When the superior and inferior incisions are made, a gently curved, blunt-tipped 26-gauge cannula on a 5ml antibiotics-filled syringe is inserted into the anterior chamber to separate the adhesion. Make sure the tip must always be upward and pressed against the endothelial surface and make a slow Z-shaped movement. When the adhesions are relieved, a curved toothed forceps is inserted from inferior incision to superior one, the plastic sheet is pulled out and sutured. Usually, hypopyon and exudate can be pushed away by injection of viscoelastic from the two sides of the plastic sheet, the thick pus or proliferative membrane covering the pupil can be taken out with curved toothless forceps / toothed forceps, an oval hole preformed in the center of the film will facilitate the removing of infectious substances in pupil area and it will not weaken the effect of the sheet to prevent extrusion of the intraocular contents. To prevent damage to donor endothelial cells, several steps would be required: first, adequate viscoelastic agent is injected between the endothelium of the graft and the sheet. The sheet is thinner and smoother than the ulcerated cornea, which has less resistance on the endothelium of donor when removed from under the implant. For the reason of convex shape, the gap between the corneal endothelium and the plastic sheet could lessen the abrasion of the endothelium while the donor is sutured directly above the flat plastic sheet. Next, care must be taken not to tying the first two stitches too tightly as normal to avoid pushing the sheet to the donor and increased abrasion of donor endothelium when removing the sheet. Finally, the plastic sheet (Fig. 2, A) was much thinner than the recipient lamellar cornea in previous technique, indicating that the space between the sheet and the graft was greater than that between the lamellar cornea and the graft.

In our cases, no serious complications associated with intraocular extrusion occurred, but it is well known that the final outcome may depend on the check-up and medication. There are still some limitations for our method. For example, the follow-up period is not long. Some patients may be lost during follow-up. Occasionally, very few patients may receive enucleation in another hospital due to recurrent infection or refractory glaucoma, and it will be difficult to know the long-term efficacy of the procedure and may affect the overall surgical outcomes. It is necessary to strengthen communication with the patient and improve their good compliance.

The plastic sheet was easily prepared with no special equipment was required. This innovative technique based on this effective, easily constructed plastic sheet facilitated the proper positioning of the anterior segment during PK for infectious keratitis patients.

## Data Availability

The data that support the findings of this study are available on request from the corresponding author.
